# Pemphigus Vulgaris With an Elevated Faecal Calprotectin Mimicking Inflammatory Bowel Disease: A Case Report

**DOI:** 10.7759/cureus.103550

**Published:** 2026-02-13

**Authors:** Abdullah Abdullah, Deborah Bertfield, Clarice Chan

**Affiliations:** 1 General Medicine, Barts Health NHS Trust - Newham Hospital, London, GBR; 2 Geriatrics, Barnet Hospital, London, GBR; 3 Internal Medicine, Barnet Hospital, London, GBR

**Keywords:** calprotectin, dermatology, faecal calprotectin, pemphigus, pemphigus vulgaris

## Abstract

We present the case of a 57-year-old woman with severe, recurrent oral ulceration and weight loss. Faecal calprotectin was markedly elevated at 1,246 µg/g, initially raising suspicion of inflammatory bowel disease with oral manifestations. However, endoscopic evaluation revealed no gastrointestinal pathology. Oral biopsy with direct immunofluorescence revealed pemphigus vulgaris.

This case illustrates two important reminders for general physicians. Firstly, approximately half of all patients with pemphigus vulgaris present with oral lesions alone, without cutaneous involvement, for months before diagnosis. Secondly, faecal calprotectin elevation can result from swallowing oral inflammatory exudate rather than bowel pathology.

Clinicians should consider autoimmune blistering disorders in patients with unexplained severe oral ulceration unresponsive to standard treatments, and interpret faecal calprotectin within the complete clinical context.

## Introduction

Pemphigus vulgaris is a rare autoimmune blistering disease characterised by intraepithelial acantholysis caused by autoantibodies against desmosomal proteins, specifically desmoglein 3 and desmoglein 1 [1]. The disease affects stratified squamous epithelia, most commonly oral mucosa and skin, with an estimated incidence of 0.1-0.5 per 100,000 population annually [2].

Clinical presentation varies considerably. Approximately 50% of patients present with exclusively oral mucosal lesions, without cutaneous involvement, a phenotype characterised by anti-desmoglein 3 antibodies [1]. These mucosal-predominant cases often experience diagnostic delays, as oral lesions may be mistaken for more common conditions such as aphthous ulceration or oral lichen planus. Diagnosis requires correlation of clinical findings with histopathology showing suprabasal acantholysis and direct immunofluorescence demonstrating intercellular IgG and C3 deposition [3].

Faecal calprotectin has become a widely used biomarker for detecting intestinal inflammation, with elevated levels (>250 µg/g) demonstrating high sensitivity and specificity for inflammatory bowel disease [4]. However, faecal calprotectin may be elevated in various gastrointestinal and extra-intestinal conditions. Its interpretation requires careful clinical correlation, particularly when upper gastrointestinal or oral pathology is present.

We present a case of mucosal-predominant pemphigus vulgaris with markedly elevated faecal calprotectin, initially suggesting inflammatory bowel disease, highlighting important diagnostic considerations for general physicians.

## Case presentation

**Figure 1:**
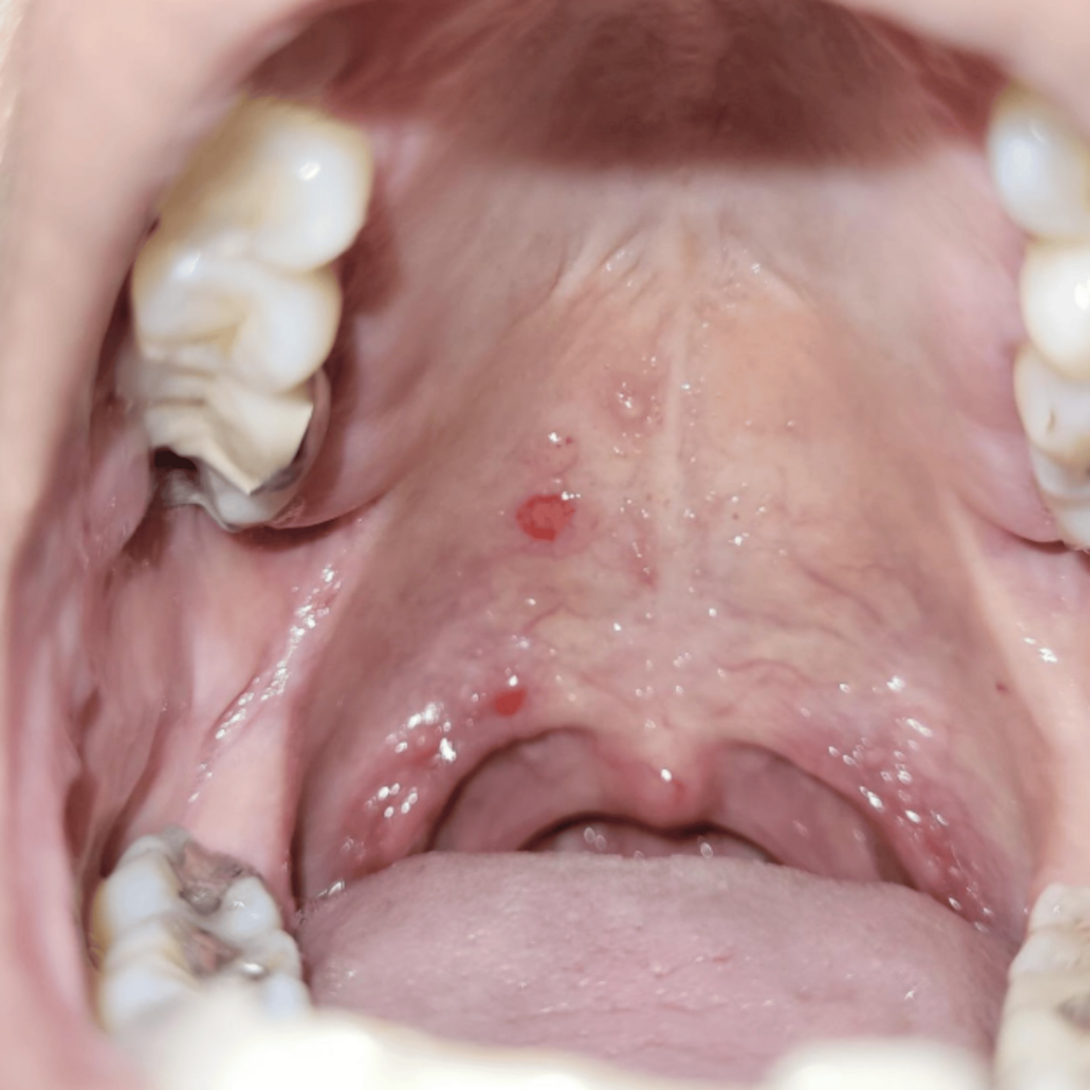
Multiple aphthous-like ulcerations involving the hard palate during early phase of disease

A 57-year-old woman with a background of pre-diabetes and eczema presented to the hospital with a 10-month history of progressive, painful oral ulceration. Her symptoms began insidiously as small aphthous-like ulcers affecting the buccal mucosa and lips, which initially resolved intermittently, as shown in Figure 1.

**Figure 2:**
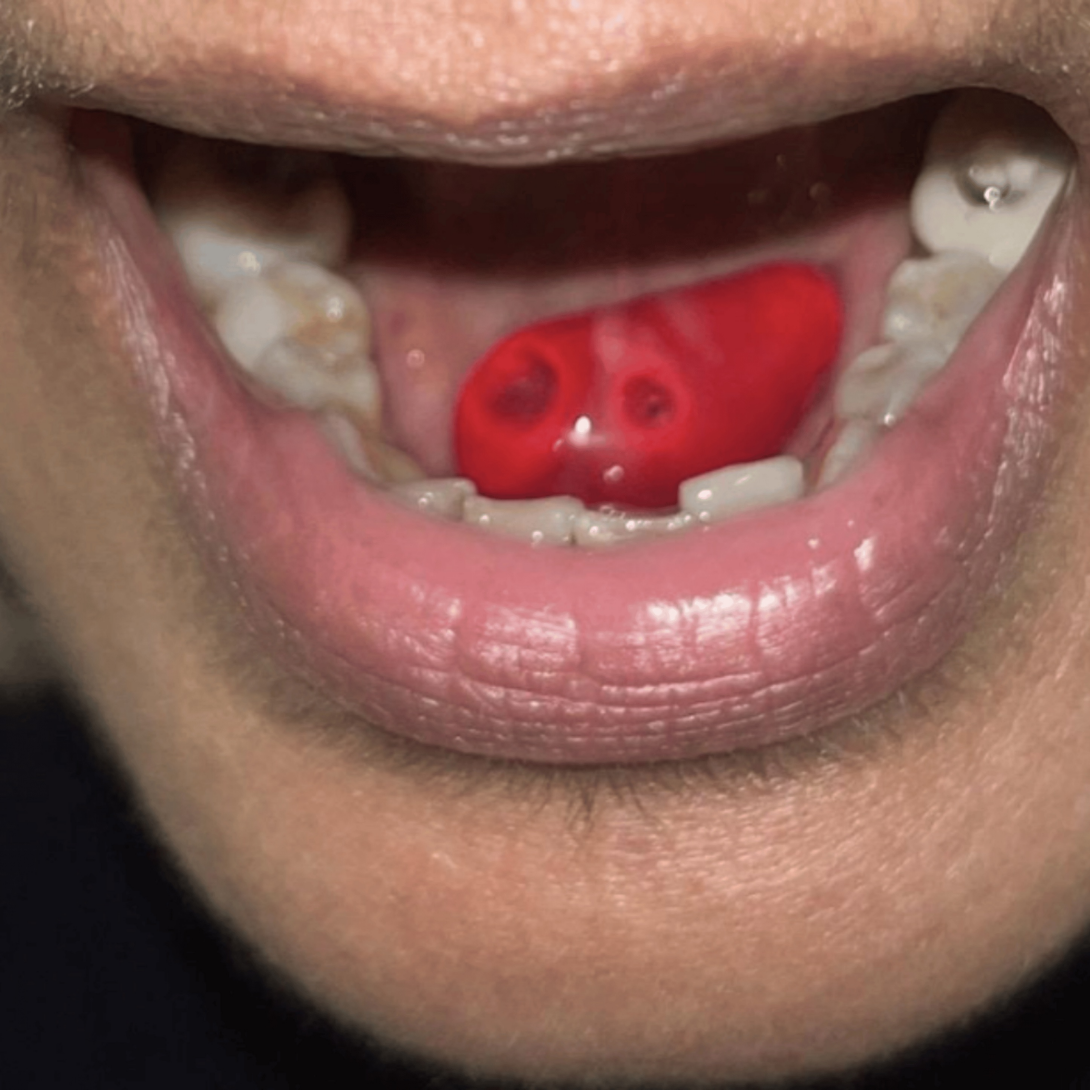
A large haemorrhagic bulla on the undersurface of the tongue

However, over subsequent months, the lesions increased in both frequency and severity. She reported that ulcers often began as fragile vesicles and bullae that rapidly ruptured, leaving painful erosions. On one occasion, she noticed a large haemorrhagic bulla beneath her tongue that subsequently broke down, as shown in Figure 2.

As the disease progressed, the oral mucosa became extensively ulcerated, involving the buccal mucosa, tongue, palate, and gingiva. The pain was so severe that it significantly impaired her ability to speak and swallow, leading to a marked reduction in oral intake and unintentional weight loss of 7-10 kg over an eight-week period.

Approximately six weeks before presentation, she developed additional mucosal symptoms. She noted bilateral conjunctival redness with mucous discharge and a painful ulcer on the right labia minora. She also described constipation with bright red rectal bleeding on wiping, though without diarrhoea, abdominal pain, or urgency. She denied any systemic symptoms, such as fever, night sweats, or cutaneous blisters elsewhere on her body.

Her medical history was notable only for pre-diabetes and eczema. There was no personal or family history of autoimmune blistering disorders or inflammatory bowel disease. She had travelled to Mauritius approximately 10 months before symptom onset but reported no associated illness. She was a non-smoker and consumed alcohol occasionally.

On examination, she appeared fatigued but was haemodynamically stable with normal vital signs. Oral cavity examination revealed diffuse mucosal erosions with fibrinous slough and contact bleeding, affecting the buccal mucosa, tongue, palate, and gingiva. The tongue was particularly tender and diffusely coated. A 1-1.5 cm painful ulcer with a clean base was present on the right labia minora. Bilateral conjunctival examination demonstrated pseudomembranous changes without corneal involvement. Notably, there were no skin lesions, no peripheral lymphadenopathy, and abdominal examination was unremarkable.

Initial laboratory investigations revealed mild neutropenia, with a neutrophil count of 1.06 × 10⁹/L (reference range 2.0-7.5 × 10⁹/L), and an elevated C-reactive protein. Iron studies demonstrated low serum iron with preserved ferritin, consistent with functional iron deficiency in the context of systemic inflammation and reduced dietary intake. Table 1 shows the results of initial blood tests on admission.

**Table 1:** Initial investigations on admission CRP: C Reactive Protein; ESR: Erythrocyte Sedimentation Rate

Test	Result	Reference Range	Interpretation
Haemoglobin	123 g/L	115-160 g/L	Mild drop from baseline
White Cell Count	2.8 × 10⁹/L	4.0-11.0 ×10⁹/L	Leukopenia
Neutrophils	1.06 × 10⁹/L	2.0-7.5 × 10⁹/L	Neutropenia
Platelets	290 × 10⁹/L	150-450 × 10⁹/L	Normal
CRP	49.5 mg/L	<5 mg/L	Inflammatory response
ESR	23 mm/hr	<30 mm/hr (1st hour)	Mild elevation
Ferritin	206-208 µg/L	13-150 µg/L	Acute phase elevation
Serum Iron	3.3 µmol/L	6-35 µmol/L	Functional iron deficiency
Vitamin D	26 nmol/L	>50 nmol/L	Deficient

Given the multi-mucosal involvement and travel history, an extensive infectious disease screen was undertaken. This included testing for human immunodeficiency virus (HIV), hepatitis B and C, syphilis serology, herpes simplex virus (HSV) and varicella zoster virus (VZV) polymerase chain reaction (PCR), cytomegalovirus (CMV) and Epstein-Barr virus (EBV) PCR, *Strongyloides* serology, *Leishmania* serology, and tuberculosis screening with interferon-gamma release assay and tissue culture. Genital and stool cultures were also performed. All infectious investigations returned negative, as shown in Table 2.

**Table 2:** Extended infectious screen results HIV: Human Immunodeficiency Virus; Hep B/Hep C: Hepatitis B and Hepatitis C viruses; HSV: Herpes Simplex Virus; VZV: Varicella Zoster Virus; CMV: Cytomegalovirus; EBV: Epstein-Barr Virus; PCR: Polymerase Chain Reaction (molecular detection of viral DNA/RNA); Quantiferon-TB Gold: Interferon-gamma release assay used to assess latent tuberculosis infection

Test	Result	Interpretation
HIV/Hep B/Hep C/Syphilis	Negative	No systemic infection
HSV/VZV PCR	Negative	Ulcers not herpetic
CMV/EBV PCR	EBV low copy only	Not clinically significant
*Strongyloides*/*Leishmania*	Negative	No travel-related infection
Quantiferon-TB Gold	Negative	Latent TB excluded

Autoimmune screening, including antinuclear antibodies (ANA), extractable nuclear antigens (ENA), anti-neutrophil cytoplasmic antibodies (ANCA), and rheumatoid factor (RF), was negative.

Of particular note, faecal calprotectin was markedly elevated at 1,246 µg/g (reference range <50 µg/g), which raised significant concern for underlying inflammatory bowel disease, particularly given the rectal bleeding and constipation. While levels >250 µg/g are typically considered highly specific for IBD, this case demonstrates that even markedly elevated values cannot be interpreted in isolation from the complete clinical picture.

Consequently, gastrointestinal evaluation was pursued. Oesophagogastroduodenoscopy demonstrated ulceration confined to the oral cavity and oropharynx, with entirely normal oesophageal, gastric, and duodenal mucosa. Flexible sigmoidoscopy was performed and was entirely normal, showing no evidence of fissures, ulceration, inflammation, or proctitis. Colonic biopsies were histologically normal, effectively excluding inflammatory bowel disease.

The complete absence of gastrointestinal pathology, despite markedly elevated faecal calprotectin, was unexpected and prompted reconsideration of the clinical picture. At this juncture, several key clinical features guided further investigation: (1) the chronicity and severity of oral ulceration unresponsive to standard aphthous treatments, (2) the presence of fragile bullae, unusual for common causes of oral ulceration, (3) conjunctival involvement suggesting a mucosal-predominant process, and (4) the complete absence of cutaneous lesions. These features raised suspicion for an autoimmune mucocutaneous disorder, specifically pemphigus vulgaris in its mucosal-predominant form.

A mucosal biopsy from the lower inner labial mucosa was then performed. Histopathological examination demonstrated intraepithelial acantholysis (loss of cohesion between keratinocytes), with basal keratinocytes remaining attached to the basement membrane in the characteristic “tombstone” pattern. Direct immunofluorescence microscopy of perilesional tissue revealed intercellular deposition of IgG and C3 within the epithelium, creating a characteristic “fish-net” or “chicken-wire” pattern. Indirect immunofluorescence serology confirmed the presence of circulating anti-desmoglein 3 IgG antibodies at a titre of 1:160 and anti-desmoglein 1 IgG antibodies at a titre of 1:40, as shown in Table 3. These findings confirmed the diagnosis of mucosal-predominant pemphigus vulgaris.

**Table 3:** Pemphigus vulgaris immunoserology results IgG: Immunoglobulin G; IIF: Indirect Immunofluorescence

Test	Result	Interpretation
Anti-Desmoglein 3 IgG	1:160	Strongly positive
Anti-Desmoglein 1 IgG	1:40	Positive
IIF Anti-Desmosomal IgG	1:320	Diagnostic titre

Following confirmation of the diagnosis, the patient was commenced on oral prednisolone at an initial dose of 30 mg daily (approximately 0.5 mg/kg, appropriate for predominantly mucosal disease), along with topical betamethasone sodium phosphate 0.5 mg mouthwash (made extemporaneously), used four times daily as an adjunctive local treatment. She was also prescribed omeprazole for gastroprotection and advised on oral hygiene measures.

She experienced rapid and marked improvement in oral pain and oral intake within the first week of treatment. The conjunctival inflammation also resolved promptly. Prednisolone was gradually tapered according to clinical response over subsequent weeks.

However, when the prednisolone dose was reduced to approximately 12.5 mg daily, she developed new cutaneous erosive lesions on the scalp and forearms, indicating disease flare and progression from purely mucosal to mucocutaneous pemphigus vulgaris. This development necessitated a steroid-sparing strategy.

Mycophenolate mofetil was initiated at 500 mg twice daily as an immunosuppressive, steroid-sparing agent, with the dose subsequently titrated to 1 g twice daily over four weeks. The prednisolone taper was then resumed at a slower rate under close monitoring. She continued topical betamethasone mouthwash as adjunctive therapy.

At follow-up appointments, the patient reported substantial improvement in oral tolerance and ability to eat. She regained approximately 5 kg of the weight she had lost. The oral mucosal lesions showed progressive healing, with a reduction in both extent and severity. The cutaneous lesions on the scalp and forearms also improved with the combination of systemic corticosteroids and mycophenolate mofetil.

She remained under regular follow-up in the dermatology department, with ongoing monitoring of disease activity, treatment side effects, and blood parameters, including full blood count, liver function, and renal function. The plan was to continue a very gradual corticosteroid taper whilst maintaining mycophenolate mofetil as the primary maintenance immunosuppressive agent.

## Discussion

This case illustrates two important lessons for general physicians evaluating patients with unexplained oral ulceration. Firstly, approximately 50% of patients with pemphigus vulgaris present with exclusively oral mucosal lesions [1]. The oral cavity may remain the only site of involvement for months, with an average delay to diagnosis of six months [2]. This “mucosal-predominant” phenotype is characterised by anti-desmoglein 3 antibodies.

Most patients eventually develop cutaneous involvement, as occurred in this case when steroids were tapered. This reflects the emergence of anti-desmoglein 1 antibodies [3]. The absence of skin lesions should not exclude pemphigus from the differential diagnosis of severe oral ulceration.

Secondly, faecal calprotectin can be markedly elevated without bowel pathology. Faecal calprotectin is widely used as a biomarker for intestinal inflammation, with levels >250 µg/g generally considered highly specific for inflammatory bowel disease [4]. However, this case demonstrates that extensive oral ulceration can cause marked elevation through swallowing inflammatory exudate. Normal endoscopic and histological findings confirmed the absence of bowel disease. Clinicians should interpret faecal calprotectin within the complete clinical context, particularly when significant upper gastrointestinal or oral pathology is present.

In unexplained severe oral ulceration unresponsive to standard treatments, biopsy with direct immunofluorescence is essential [5]. The characteristic intercellular IgG deposition with suprabasal acantholysis is diagnostic of pemphigus vulgaris.

Early recognition enables prompt treatment. First-line therapy consists of systemic corticosteroids (typically prednisolone 0.5-1 mg/kg/day), often combined with steroid-sparing immunosuppressive agents, such as azathioprine or mycophenolate mofetil, for long-term disease control. Rituximab has emerged as an effective option for refractory cases [5-7]. A comprehensive summary of treatment options is provided in Table 4.

**Table 4:** Treatment options for pemphigus vulgaris

Treatment Category	Agent	Typical Dose	Role
First-line	Prednisolone	0.5-1 mg/kg/day	Initial disease control
Steroid-sparing agents	Azathioprine	1-3 mg/kg/day	Long-term maintenance
	Mycophenolate mofetil	1 g twice daily	Alternative to azathioprine
Biologic therapy	Rituximab	1 g on days 1 and 15	Refractory or severe disease
Adjunctive	IVIg	2 g/kg per cycle	Refractory disease
Topical	Betamethasone mouthwash	0.5 mg in 10 mL	Adjunct for oral lesions

## Conclusions

This case highlights the importance of considering mucosal-predominant pemphigus vulgaris in patients presenting with severe oral ulceration in the absence of cutaneous involvement. Markedly elevated faecal calprotectin levels may occur secondary to swallowed inflammatory exudate from extensive oral mucosal disease, and should not be assumed to represent primary gastrointestinal pathology in this context. Direct immunofluorescence microscopy, supported by anti-desmoglein serology, remains essential for establishing the diagnosis of pemphigus vulgaris. Clinicians should remain vigilant, as mucosal-predominant disease may progress to mucocutaneous involvement over time. Early recognition and timely initiation of steroid-sparing immunosuppressive therapy are crucial to achieve disease control while minimising cumulative corticosteroid exposure.
